# Early vs. Late Readmission following Percutaneous Coronary Intervention: Predictors and Impact on Long-Term Outcomes

**DOI:** 10.3390/jcm12041684

**Published:** 2023-02-20

**Authors:** David Eccleston, My-Ngan Duong, Enayet Chowdhury, Nisha Schwarz, Christopher Reid, Danny Liew, Andre Conradie, Stephen G. Worthley

**Affiliations:** 1 Department of Medicine, University of Melbourne, Parkville, VIC 3050, Australia; 2 GenesisCare, Leabrook, SA 5068, Australia; 3 School of Public Health, Curtin University, Perth, WA 6845, Australia; 4 Adelaide Med School, Adelaide, SA 5000, Australia; 5 Cardiology Department, Friendly Society Private Hospital, Bundaberg, QLD 4670, Australia; 6 North Shore Cardiology, St Leonards, NSW 2065, Australia

**Keywords:** percutaneous coronary intervention, unplanned readmission, MACE, death, registry

## Abstract

Background: Readmissions within 1 year after percutaneous coronary intervention (PCI) are common (18.6–50.4% in international series) and a burden to patients and health services, however their long-term implications are not well characterised. We compared predictors of 30-day (early) and 31-day to 1-year (late) unplanned readmission and the impact of unplanned readmission on long-term clinical outcomes post-PCI. Methods: Patients enrolled in the GenesisCare Cardiovascular Outcomes Registry (GCOR-PCI) from 2008 to 2020 were included in the study. Multivariate logistic regression analysis was performed to identify predictors of early and late unplanned readmission. A Cox proportion hazards regression model was used to explore the impact of any unplanned readmission during the first year post-PCI on the clinical outcomes at 3 years. Finally, patients with early and late unplanned readmission were compared to determine which group was at the highest risk of adverse long-term outcomes. Results: The study comprised 16,911 consecutively enrolled patients who underwent PCI between 2009–2020. Of these, 1422 patients (8.5%) experienced unplanned readmission within 1-year post-PCI. Overall, the mean age was 68.9 ± 10.5 years, 76.4% were male and 45.9% presented with acute coronary syndromes. Predictors of unplanned readmission included increasing age, female gender, previous CABG, renal impairment and PCI for acute coronary syndromes. Unplanned readmission within 1 year of PCI was associated with an increased risk of MACE (adjusted HR 1.84 (1.42–2.37), *p* < 0.001) and death over a 3-year follow-up (adjusted HR 1.864 (1.34–2.59), *p* < 0.001) compared with those without readmission within 1-year post-PCI. Late compared with early unplanned readmission within the first year of PCI was more frequently associated with subsequent unplanned readmission, MACE and death between 1 and 3 years post-PCI. Conclusions: Unplanned readmissions in the first year following PCI, particularly those occurring more than 30 days after discharge, were associated with a significantly higher risk of adverse outcomes, such as MACE and death at 3 years. Strategies to identify patients at high risk of readmission and interventions to reduce their greater risk of adverse events should be implemented post-PCI

## 1. Introduction

Over the past 20 years, percutaneous coronary intervention (PCI) has become the predominant mode of revascularisation for coronary artery disease in Europe, the United States and Australia [1,2,3]. However, despite advances in technology and clinical practice, unplanned readmissions to the hospital after PCI continue to significantly impact patient outcomes, health systems and the economy [4,5]. Readmission rates vary from 4.7–15.6% at 30 days, and from 18.6–50.4% at 1 year in international series [5,6,7,8]. Unplanned readmissions may reflect incomplete or lower-quality hospital care; poor coordination of services post-discharge; or an adverse outcome from complications, such as bleeding from dual anti-platelet therapy, which was demonstrated to increase mortality after PCI [9]. They represent a potentially avoidable cost and are also increasingly used as a quality metric with financial penalties for hospitals in both the USA and the UK, where readmission rates exceed certain benchmarks [9,10,11].

The causes of unplanned readmission beyond 30 days after PCI and the long-term implications of such readmissions are poorly characterised. Understanding these is crucial to reducing the risk of readmission and aid with the planning of healthcare services, as the only intervention shown to reduce readmissions is to target specific causes [12]. These questions can be informed by utilising “real-world” data from clinical quality registries that document outcomes after PCI. However, resource constraints restricted previous analyses’ use of long-term outcome data obtained via linkage to administrative datasets, a process that is subject to potential errors, including patient under-reporting of readmissions [13]. Therefore, we analysed prospectively collected long-term PCI outcome data from the large multi-centre GenesisCare Cardiovascular Outcomes Registry (GCOR) to examine the rates and predictors of unplanned readmission at 30 days, 1 year and 3 years after PCI, and then contrast these predictors to assess the potential clinical impact of earlier compared with later readmission after PCI.

## 2. Methods

We undertook a retrospective cohort study of patients who underwent PCI between 1 November 2008 and December 2020 and who had at least 1 year of follow-up in the GCOR. The GCOR was described previously [14]. Briefly, demographic, clinical, procedural, in-hospital and long-term outcome data are recorded on electronic case report forms using standardised definitions for all fields. All PCI cases performed in 12 private and 2 public hospitals by GenesisCare interventionalists across Australia were enrolled in the GCOR registry and were included in the study; all patients enrolled were eligible with no exclusion criteria. Thirty-day and annual follow-ups are performed by research coordinators at each site [15,16]. Enrolling sites are independently audited by investigators not affiliated with that institution. Written Human Research Ethics Committee (HREC) approval was obtained for the collection of patient data and follow-up prior to participation in other aspects of the GCOR, such as PCI and device implantation (Bellberry HREC Eastwood, S.A. 5063 Australia).

The registry is coordinated by the GCOR Interventional Working Group in collaboration with the Monash University Centre for Clinical Research Excellence in Therapeutics, which jointly developed the eCRF. The Centre meets standards relating to the use of paperless records under the Good Clinical Practice regulations and complies with the National E-Health Transition Authority (NEHTA) standard of reporting and storing data [17]. The systems and processes regarding privacy and data protection comply with relevant Health Records and Information Privacy Acts and Information Privacy Principles. There was no external source of funding for the study.

## 3. Ethics and Informed Consent

Each centre received written Human Research Ethics Committee (HREC) approval for the collection of patient data and follow-up prior to participation (Bellberry HREC Eastwood, S.A.); stored data was de-identified. An opt-out consent process was employed following previous registry practice [14].

## 4. Data Collection

Data collection was previously described [14]. Detailed information on all readmissions and patient survival after PCI was collected during follow-up visits. Unplanned cardiac readmission included admission for any of the following conditions: myocardial infarction (MI), unplanned PCI/coronary artery bypass graft surgery (CABG), major bleeding, angina, cerebrovascular accident (CVA)/stroke, heart failure and arrhythmia.

## 5. Statistics

We used descriptive analysis (frequency/sample proportions or mean with standard deviations) to summarise the patients’ baseline demographic and clinical characteristics, risk factors and procedural characteristics by unplanned readmission status within 30 days post-PCI (early), or between 31 and 365 days (late) post-PCI. Student’s *t*-test, ANOVA and chi-square test were used to compare the distributions of patients’ characteristics, including procedural and/or risk factors by readmission status for both periods. Multivariate logistic regression analysis was performed to determine possible predictors of unplanned readmission within 30 days, 31 days–1 year and for the overall group with unplanned readmission at any point within 1 year. All variables that were *p* ≤ 0.1 on univariate logistic regression analysis, including particularly gender, smoking, diabetes, hypertension, hypercholesterolaemia, heart failure, prior PCI, myocardial infarction, CABG and stroke, were included in the multiple logistic regression model.

A Cox proportional hazard regression model was used to compare the clinical outcomes over 3 years between those who experienced unplanned readmission within 1 year of PCI and those who did not. The model was adjusted for admission diagnosis, history of myocardial infarction, coronary revascularisation, valvular surgery, heart failure, diabetes mellitus and smoking status. Propensity score matching was performed using data from 1198 eligible patients of the 1422 patients with unplanned readmission with matched controls based on the criteria of age, male gender, diabetes, hypertension, heart failure anytime, previous myocardial infarction or CABG, renal impairment, ACS or multivessel disease. Further, we explored and compared the impact of early and late unplanned readmission on future clinical outcomes observed between 1 and 3 years, including further unplanned readmission, MACE (death, MI, repeat PCI and stroke) and death, using a Cox proportional hazard regression model. All statistical analyses were performed using Stata 17.0 for Windows. A *p*-value < 0.05 was considered to be statistically significant.

## 6. Results

The GCOR-PCI cohort comprised 16,911 patients who underwent PCI during the study period. Of the 13,877 patients who completed their 1-year follow-up, 1442 patients (8.5%) experienced unplanned readmission within the first year post-PCI. The proportion of missing data was <1% for all variables. Overall, the mean age was 68.9 ± 10.5 years, 76.4% were male and 1.4 ± 0.6 (mean ± SD) stents per lesion were implanted.

We observed 403 unplanned readmissions within the first 30 days post-PCI and 1071 with unplanned readmission between 31 and 365 days post-PCI. Unplanned cardiac readmission occurred for multiple reasons. The major reasons for unplanned cardiac readmissions in both periods were angina, followed by unplanned PCI, arrhythmia and myocardial infarction (Supplementary Table S1). Unplanned PCI was more frequently a cause of unplanned readmission in the late vs. early group (OR 2.52 (1.84–3.45), *p* < 0.001).

Overall, patients with unplanned readmission within the first year were older (69.4 ± 10.9 vs. 68.8 ± 10.5 years, *p* = 0.047); more likely to be female (26.9% vs. 23.7%, *p* = 0.002); have hypercholesterolaemia (88.2% vs. 86.3%, *p* = 0.048); and have a history of heart failure, previous MI, PCI, peripheral vascular disease, cerebrovascular disease, CABG, atrial fibrillation (all *p* < 0.001), chronic kidney disease (*p* = 0.002) or present with an acute coronary syndrome (51.4% vs. 45.5% *p*< 0.001) than those who did not experience readmission.

Table 1 depicts a comparison of the baseline clinical characteristics between patients with early (≤30 days) or late (31–365 days) unplanned readmission post-PCI.

The procedural characteristics differed significantly between patients that experienced unplanned readmission and those who did not. Complex lesion morphology was more frequent in those with unplanned readmission (B2/C 51.6% vs. 45.2%, *p* < 0.001; chronic total occlusion 5.8% vs. 4.8%, *p* = 0.043) compared with no readmission. Similarly, the presence of multivessel disease (55.6% vs. 44.3%, *p* < 0.001) and procedural failure (7.0% vs. 3.1%, *p* < 0.001) were more commonly associated with unplanned readmission. Patients requiring peri-procedural CABG (2.6% vs. 0.7%, *p* < 0.001) or experiencing major bleeding (2.2% vs. 1.5%, *p* = 0.032) were more likely to experience unplanned readmission.

Table 2 compares the procedural characteristics between patients with unplanned readmission within either the first 30 days or between 31 and 365 days post-PCI.

Table 3 describes the medications at discharge post-PCI. There were no significant differences in the use of dual anti-platelet therapies, statins, B-blockers or ACEI/ARB between patients that experienced early or late unplanned readmission.

## 7. Predictors of Unplanned Readmission

Overall, a cohort multivariate logistic regression analysis revealed several characteristics predictive of unplanned readmission within 1 year of PCI. These included female sex (OR 1.31, 95% CI 1.07–1.60, *p* = 0.001), previous CABG (OR 1.6, 95% CI 1.18–2.17, *p* = 0.03) and atrial fibrillation (OR 1.84, 95% CI 1.47–2.29, *p* < 0.001)

Patients with renal dysfunction were also at increased risk of readmission with a strong trend towards significance (OR 1.40, 95% CI 1.00 to 1.96, *p* = 0.05). Maximum stent diameter, which is a surrogate for vessel size, was also inversely associated with the increased risk of unplanned readmission (OR 0.79, 95% CI 0.66–0.96, *p* = 0.02). The risk of unplanned readmission was significantly increased if the target vessel for PCI was the LAD (OR 1.24, 95% CI 1.00–1.53, *p* = 0.048) or a bypass graft (OR 1.77, 95% CI 1.06–2.95, *p* = 0.03).

Treatment for in-stent stenosis was also associated with a greater likelihood of readmission, with a strong trend towards significance (OR 1.49, 95% CI 1.00 to 2.23, *p* = 0.05).

## 8. Predictors of Early vs. Late Unplanned Readmission

There were marked differences in the predictors of unplanned readmission to a hospital between those readmitted ≤30 days vs. those readmitted between 31 days to 1 year following PCI after an adjustment for baseline characteristics (Table 4).

Female patients were at an even greater risk of early vs. late unplanned readmission in the first year post-PCI. Similarly, patients with a multivessel disease were at a greater risk of early vs. late readmission.

Late unplanned readmission was more common in younger patients; those with an established vascular disease (peripheral vascular disease, CABG, in-stent restenosis; and those with unstable coronary syndromes, LAD disease, a small vessel diameter and who were not discharged on dual anti-platelet therapy with a P2Y12 inhibitor.

## 9. Long-Term Outcomes

Overall, unplanned readmission at any time within 1 year of PCI was associated with a significantly increased risk of all-cause mortality (HR 2.50, 95% CI 1.67 to 3.75, *p* < 0.001) at 2 years post-PCI compared with those who did not experience any unplanned cardiac readmission in the first year

**Figure 1:**
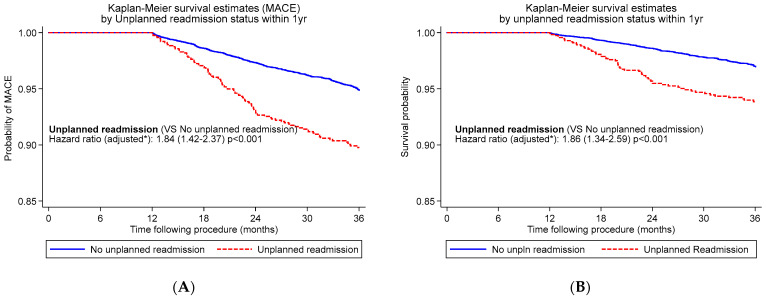
K-M curves for 3-year MACE (**A**) and survival (**B**) for patients with unplanned readmission within 1 year following PCI. * Adjusted for age, male sex, diabetes, hypertension, family history of heart failure, previous myocardial infarction, previous coronary artery bypass grafting, renal impairment, PCI presentation and multivessel disease.

Similarly, unplanned readmission within 1 year of PCI was associated with a significantly increased risk of further all-cause readmission (18.5% vs. 13.2% *p* = 0.005) in the interval between 2 and 3 years post-PCI. There was a significantly increased risk of MACE between year 1 and 3 following PCI in patients experiencing unplanned readmission 1 year post-PCI compared with those who were not readmitted (adjusted HR 1.84 (1.42–2.37), *p* < 0.001) (Figure 1A).

Further, there was a significantly increased risk of death between years 1 and 3 following PCI in patients with unplanned readmission within 1 year of PCI compared with those who were not readmitted (adjusted HR 1.86 (1.34–2.59), *p* < 0.001) (Figure 1B). We repeated the above analyses in a propensity-matched sample and observed similar findings.

## 10. Impact of Early vs. Late Readmission on Long-Term Outcomes

Of the total 1474 unplanned readmissions (403 early and 1071 late) within the first year following PCI, after the exclusion of patients with multiple readmissions, death within 1 year and no follow-up beyond 1 year, 1047 patients with unplanned readmission (206 early, 841 late) were available for comparison of potential differences between early and late readmission regarding long-term clinical outcomes between 1 and 3 years post-PCI.

**Figure 2:**
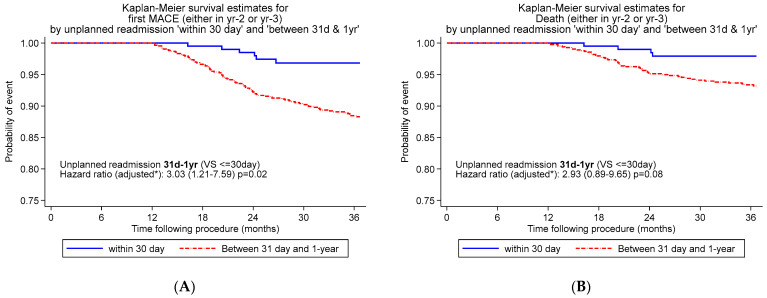
K-M curves for 3-year MACE (**A**) and survival (**B**) for patients with early (<30 days) vs. late (31 days–1 year) unplanned readmission following PCI. * Adjusted for age, male sex, diabetes, hypertension, family history of heart failure, previous myocardial infarction, previous coronary artery bypass grafting, renal impairment, PCI presentation and multivessel disease.

Late unplanned readmission was a stronger predictor of subsequent unplanned readmission between 1 and 3 years than early readmission (18.9% vs. 10.7%, *p* = 0.005). Similarly, late unplanned readmission was more commonly associated with MACE (10.8% vs. 2.9%, *p* < 0.001) at 3 years post-PCI than early readmission (Figure 2A).

Further, late unplanned readmission was more commonly associated with all-cause mortality (6.3% vs. 1.9%, *p* = 0.01) than early readmission between 1 and 3 years post-PCI (Figure 2B).

## 11. Discussion

This large multi-centre study demonstrated that patients that underwent PCI in Australian private hospitals had a relatively low rate (8.5%) of unplanned readmission at 1 year. Nearly half (3.4%) of all unplanned readmissions within the first year occurred in the first 30 days, with the risk decreasing thereafter. Many unplanned readmissions were non-cardiac in aetiology, with 46.4% of readmissions being primarily for a cardiac diagnosis. Independent predictors of 1-year unplanned readmission were increased age, female sex, left ventricular dysfunction, peripheral vascular disease, prior CABG, chronic kidney disease, undergoing PCI for ACS, complex lesion morphology and chronic total occlusion.

Performance measures for PCI often include the rate of unplanned readmission at 30 days; however, the continuing burden and clinical impact of readmission beyond this timeframe to 6 and even 12 months post-PCI are being increasingly recognised [18,19]. The unplanned 1-year readmission rate in this cohort was lower than that reported overseas at 6 months or 1-year follow-up, and similar to many reported 30-day readmission rates [8,20,21,22].

There are several potential explanations for the lower unplanned readmission rate in this series compared with international reports. Some studies that reported higher unplanned readmission rates at 1 year or similar rates at 30 days included data from only 1 or 2 hospitals, whereas this study included 14 private and 2 public hospitals across Australia and was, therefore, more representative of practice from a national perspective [19,21]. Other series included populations from countries with nationalised health services, as well as combinations of insured and uninsured patients as in the U.S., raising the possibility that the lower rate of unplanned readmission in this report was due to differences in baseline characteristics between their populations and this cohort. However, patient and procedural characteristics were similar to reports from studies such as the Swedish Web-System for Enhancement and Development of Evidence-Based Care in Heart Disease Evaluated According to Recommended Therapies (SWEDEHEART) and NCDR Cath-PCI [14,23,24]. Further, characteristics that are known predictors of readmission were in similar proportion to overseas studies, with, for example, nearly half of the patients undergoing PCI for ACS [7]. Previous studies suggested that readmission rates are lower for patients having their index PCI at a private hospital, with similar findings for readmission for heart failure or after orthopaedic surgery [25].

Importantly, half of all unplanned cardiac readmissions in this study were due to chest pain, which included some patients with angina; however, many had atypical or non-specific symptoms without a cardiac diagnosis. Overseas reports demonstrated that most such readmissions are due to low-risk chest pain that does not require any intervention [4]. Previous studies demonstrated that these readmissions may be potentially preventable by developing nurse-led chronic disease management programmes to improve medication compliance and patient education, with early post-PCI follow-up. Although these programmes can reduce the risk of readmissions and improve compliance, self-care behaviour and quality of life in patients with heart failure, their use is less widespread among patients undergoing PCI [5,26]. Therefore, over the past year, we have increasingly enrolled PCI patients into nurse-led chronic disease management programs with pre-discharge education where possible and in all early post-discharge reviews in conjunction with the cardiologist. These nurse-led programmes may also provide for early identification of patients with non-modifiable risk factors, such as older age, female sex, chronic kidney disease and other co-morbidities, to direct a more intensive review in an effort to reduce their risk of readmission from both PCI-related conditions and also non-cardiac conditions that are more common in those with multiple co-morbidities, such as patients aged over 60 [5,27].

The marked differences in the predictors of unplanned readmission to hospital between those readmitted early (≤30 days) compared with those experiencing late (31 days to 1 year) readmission may relate to differing mechanisms, Early readmission may be more related to certain patient- or procedure-specific issues. Female gender was a stronger predictor of early than late unplanned readmission, perhaps reflecting the fact that gender-specific differences in presentation or treatment are at play. It is well documented that females are less likely to receive early intervention for acute coronary syndromes than males and have higher rates of complications, including bleeding following PCI, that may prompt an earlier presentation to a hospital. Similarly, multivessel disease predicted early but not late readmission, possibly due to ongoing symptoms of ischaemia requiring further presentations or additional intervention.

In contrast, factors that predicted late unplanned readmission appeared to be more lesion- and treatment-specific, including PCI for in-stent LAD or bypass graft lesion site and small vessel diameter, all of which may be associated with higher risks of restenosis and recurrent ischaemic events. Failure to be discharged on a P2Y12 inhibitor (which may occur in some who receive dual anti-thrombin therapy with aspirin and concomitant anticoagulant therapy for AF, for example) also predicted late unplanned readmission, perhaps due to an increased risk of ischaemic events.

This study was the first to our knowledge to identify unplanned readmission at any time within 1 year of PCI as a significant independent predictor of long-term MACE and mortality after PCI. Several characteristics were identified as risk factors for long-term mortality after PCI, including older age, extreme obesity, multivessel disease, a lower ejection fraction, unstable hemodynamic state or shock, several comorbidities (cerebrovascular disease, peripheral vascular disease, congestive heart failure, chronic obstructive pulmonary disease, diabetes mellitus and renal failure) and a history of coronary artery bypass graft surgery [28]. However, there is little evidence showing whether unplanned readmission itself is an independent risk factor for long-term adverse outcomes. One report demonstrated that 60-day readmissions were associated with a higher risk of all-cause death during a 2-year follow-up; however, they were not independent predictors of MACE or all-cause death [29]. However, this was a single-centre study, and the sample size of 1193 patients may have been too small to detect the true effect of unplanned readmission on outcomes. The main predictors of mortality in that study were chronic kidney disease and multivessel disease, which were accounted for in the regression model in our study. In one study, a stepwise increase in the risk of both cardiac and non-cardiac 30-day readmissions was observed with each extra admission between 1 and ≥4 admissions in the 12 months prior to PCI (*p* < 0.001). Pre-discharge identification and early follow-up of patients that were deemed to be high-risk for readmission following PCI with improved in-hospital education regarding cardiac symptoms and medication compliance was previously shown to be effective at reducing 30-day readmission rates [12].

The prospective GCOR study had both similarities and some important differences from other reports regarding readmission, particularly those using administrative datasets. The U.S. National Readmission Database, for example, is constructed in a format that precludes linkage between years, and thus, it is possible that the same patient may appear in more than 1 year, and it is not possible to follow patients across years, whereas patients enrolled in GCOR have a unique identifier to avoid multiple counting. Further, due to the nature of the annualised data, each patient in the NRD has a maximum follow-up of only 1 year, with the numbers at risk decreasing with time, whereas patients enrolled in the GCOR are followed prospectively and annually from the time of PCI. Third, the NRD cannot determine regional variations within the dataset, and hence, results may be generalisable only to the U.S. healthcare system; the GCOR includes urban/rural and locality data and was used to demonstrate region-based variations in care and outcomes [30]. Finally, the NRD excludes patients admitted during December, outpatient PCI and several fields for the last 6 months of the year, and thus, seasonal effects may not be captured, whereas the GCOR enrols patients continuously throughout the year and includes all PCIs, including day cases. One additional limitation of using administrative data, such as that from NRD, is the uncertainty in the classification of nonspecific chest pain as a reason for readmission. This code is derived from codes for chest pain, unspecified, precordial pain and other chest pain, and in one analysis, it was classified as noncardiac; however, there is the potential that the pain coded using these terms may have been cardiac in origin, leading to an underestimation of the number of unplanned cardiac readmissions; therefore, in this study, all presentations with chest pain were included as a cardiac cause of readmission, and were the most common cause of unplanned readmission.

The potential limitations of this study were the fact that only a proportion of Australian hospitals were included, and only two public hospitals were represented; all patients treated in these two public hospitals were managed by GenesisCare cardiologists. Patients treated in private hospitals may differ from those managed in the public sector. However, our data demonstrated patients and lesion characteristics comparable to those in international series, such as SWEDEHEART and NCDR Cath-PCI. Patients managed in private hospitals may receive different care from those in public hospitals, and hence, these data may not reflect practice and outcomes in non-participating hospitals.

Further, even though we were able to adjust for a variety of variables and comorbidities, there is still the risk of residual confounding, as the study was observational in nature and the GCOR does not capture measures of frailty that may affect PCI outcomes [31].

## 12. Conclusions

Unplanned readmission in the first year after PCI was independently associated with a significantly higher risk of adverse outcomes, such as MACE and death at 3 years. Patients experiencing late (31–365 days) compared with early (≤30 days) unplanned readmission in the first year post-PCI were at even greater risk of further unplanned readmission, MACE and death at 3 years following PCI. Strategies to identify patients at high risk of readmission and interventions to reduce their greater risk of adverse events should be implemented post-PCI.

## Figures and Tables

**Table 1:** Baseline clinical characteristics.

	Unplanned Readmission ≤30 Days			Unplanned Readmission 31 Days–1 Year		
Characteristics	No	Yes	*p*-Value	No	Yes	*p*-Value
N (%)	16,237	403		12,806	1071	
Age, yr, mean (±SD)	68.8 (10.5)	69.7 (11.5)	0.10	68.5 (10.5)	69.3 (10.6)	0.02
Male	12,383 (76.3%)	291 (72.2%)	0.045	9842 (76.9%)	782 (73.0%)	0.004
Diabetes	4007 (24.7%)	88 (21.8%)	0.18	3071 (24.0%)	297 (27.7%)	0.005
Hypertension	11,683 (72.0%)	315 (78.2%)	0.01	9225 (72.0%)	835 (78.0%)	<0.001
Hypercholesterolaemia ^1^	13,083 (80.6%)	332 (82.4%)	0.36	10,405 (81.3%)	899 (83.9%)	0.06
CAD family history	5738 (35.3%)	125 (31.0%)	0.06	4671 (36.5%)	394 (36.8%)	0.91
Smoking ever	8282 (51.0%)	212 (52.6%)	0.87	6730 (52.6%)	568 (53.0%)	0.79
BMI, kg/m^2^, mean (±SD)	29.0 (5.0)	28.7 (5.2)	0.29	28.9 (5.0)	28.9 (4.7)	0.76
Heart failure	1079 (6.6%)	48 (11.9%)	<0.001	831 (6.5%)	103 (9.6%)	<0.001
Prior MI	3533 (21.8%)	106 (26.3%)	0.04	2789 (21.8%)	285 (26.6%)	<0.001
Prior PCI	5271 (32.5%)	140 (34.7%)	0.30	4106 (32.1%)	410 (38.3%)	<0.001
PVD	1161 (7.2%)	37 (9.2%)	0.14	913 (7.1%)	109 (10.2%)	<0.001
Prior stroke/TIA	1036 (6.4%)	32 (7.9%)	0.24	819 (6.4%)	112 (10.5%)	<0.001
Prior CABG	1678 (10.3%)	61 (15.1%)	0.002	1315 (10.3%)	201 (18.8%)	<0.001
Prior AF	1791 (14.2%)	70 (22.2%)	<0.001	1282 (13.1%)	180 (22.8%)	<0.001
Renal failure ^2^	828 (5.8%)	36 (10.1%)	<0.001	623 (5.5%)	69 (7.2%)	0.02
Clinical presentation			<0.001			<0.001
STEMI	1113 (6.9%)	30 (7.4%)		912 (7.1%)	51 (4.8%)	
NSTEMI	3452 (21.3%)	128 (31.8%)		2720 (21.2%)	218 (20.4%)	
UAP	2479 (15.3%)	72 (17.9%)		1998 (15.6%)	232 (21.7%)	
Elective	8394 (51.7%)	160 (39.7%)		6765 (52.8%)	537 (50.1%)	
Cardiogenic shock	58 (0.4%)	1 (0.2%)	0.72	44 (0.3%)	2 (0.2%)	0.39

^1^ Hypercholesterolamia was defined as either (a) cholesterol level >5.2 and/or (b) receiving medication. ^2^ Renal impairment was defined as either (a) S. creatinine >2 mg/dL and/or (b) having renal failure/receiving treatment. CAD—coronary artery disease; BMI—body mass index; MI—myocardial infarction; PVD—peripheral vascular disease; TIA—transient ischaemic attack; AF—atrial fibrillation; STEMI—ST elevation MI; NSTEMI—non-ST-elevation MI; UAP—unstable angina.

**Table 2:** Procedural characteristics.

	Unplanned Readmission ≤30 Days			Unplanned Readmission 31 Days–1 Year		
Characteristics	No	Yes	*p*-Value	No	Yes	*p*-Value
N (%)	16,237	403		12,806	1071	
Lesion access site			0.54			<0.001
Brachial	48 (0.3%)	0 (0.0%)		40 (0.3%)	3 (0.3%)	
Radial	7177 (44.2%)	178 (44.2%)		5125 (40.0%)	341 (31.8%)	
Femoral	8824 (54.3%)	222 (55.1%)		7580 (59.2%)	720 (67.2%)	
Lesion type			0.10			<0.001
De novo	13,496 (83.1%)	332 (82.4%)		10,914 (85.2%)	891 (83.2%)	
In stent restenosis	748 (4.6%)	29 (7.2%)		579 (4.5%)	88 (8.2%)	
Restenosis	90 (0.6%)	3 (0.7%)		50 (0.4%)	6 (0.6%)	
Other	1903 (11.7%)	39 (9.6%)		1263 (9.7%)	86 (8.0%)	
ACC/AHA morphology			0.85			0.88
A	1834 (14.1)	50 (15.2%)		1452 (13.4%)	118 (12.9%)	
B1	4528 (34.7%)	113 (34.2%)		3846 (35.6%)	323 (35.3%)	
B2/C	6685 (51.2%)	167 (50.6%)		5511 (50.9%)	472 (51.6%)	
Target vessel			0.12			<0.001
RCA	4312 (26.6%)	107 (26.6%)		3480 (27.2%)	248 (23.2%)	
LMCA	227 (1.4%)	8 (2.0%)		171 (1.3%)	17 (1.6%)	
LAD	6025 (37.1%)	127 (31.5%)		4796 (37.5%)	385 (35.9%)	
LCx	2960 (18.2%)	84 (20.8%)		2346 (18.3%)	205 (19.1%)	
Bypass	400 (2.5%)	14 (3.5%)		312 (2.4%)	61 (5.7%)	
Other	2313 (14.2%)	63 (15.6%)		1701 (13.3%)	155 (14.5%)	
Total occlusion	664 (4.6%)	19 (5.1%)	0.61	532 (4.5%)	53 (5.3%)	0.29
Multivessel disease	7258 (44.7%)	202 (50.1%)	0.03	5633 (44.0%)	499 (46.6%)	0.12
Bifurcation lesion	1449 (10.0%)	43 (11.7%)	0.29	1139 (9.8%)	105 (10.5%)	0.45
Stent length, mm (mean ± SD)	19.2 (6.7)	18.5 (6.7)	0.04	19.1 (6.6)	18.8 (6.4)	0.26
Stent diam., mm (mean ± SD)	3.0 (0.5)	3.0 (0.5)	0.54	3.0 (0.5)	2.9 (0.5)	<0.001
Major bleeding (BARC 3–5)	50 (0.3%)	2 (0.5%)	0.50	47 (0.4%)	0 (0.0%)	0.047

RCA—right coronary artery; LMCA—left main coronary artery; LAD—left anterior descending artery; LCX—left circumflex artery; SD—standard deviation.

**Table 3:** Discharge medications.

	Unplanned Readmission ≤30 Days			Unplanned Readmission 31 Days–1 Year		
Characteristics	No	Yes	*p*-Value	No	Yes	*p*-Value
N (%)	16,237	403		12,806	1071	
Aspirin	15,437 (96.5%)	382 (96.7%)	0.88	12,326 (97.1%)	1021 (96.0%)	0.06
P2Y12 *	14,498 (90.5%)	369 (92.9%)	0.11	11,653 (91.7%)	990 (93.2%)	0.08
Statin	14,647 (93.1%)	354 (91.0%)	0.18	11,796 (93.6%)	973 (92.1%)	0.15
Statin contra-indicated	112 (0.7%)	5 (1.2%)		86 (0.7%)	8 (0.7%)	
Beta-blocker	8983 (55.3%)	219 (54.3%)	0.55	7270 (56.8%)	607 (56.7%)	0.99
ACEI/ARB	10,625 (65.4%)	268 (66.5%)	0.48	8567 (66.9%)	714 (66.7%)	0.72

* Clopidogrel/prasugrel/ticagrelor; ACEI—angiotensin converting enzyme inhibitors; ARB—angiotensin II receptor blockers.

**Table 4:** Multivariate predictors of unplanned readmission ≤12 months post-PCI.

Characteristic	Unplanned Readmission ≤30 Days			Unplanned Readmission 31 Days–1 Year		
	Odds Ratio	95% CI	*p*-Value	Odds Ratio	95% CI	*p*-Value
**Clinical**						
Age (years)	1.00	0.98–1.01	0.55	0.99	0.98–1.0	0.046
Male	0.70	0.54–0.96)	0.03	0.80	0.64–0.98	0.03
Diabetes mellitus	-	-	-	0.94	0.76–1.16	0.58
Hypertension	1.13	0.80–1.60	0.47	1.22	0.97–1.52	0.09
Hypercholesterolemia	-	-	-	1.11	0.84–1.46	0.47
Heart failure	1.37	0.82–2.20	0.20	1.08	0.77–1.50	0.67
Previous MI	0.99	0.70–1.40	0.95	0.93	0.74–1.18	0.56
Previous PCI	-	-	-	0.97	0.78–1.21	0.79
Peripheral vascular disease	-	-	-	1.37	1.01–1.85	0.04
Previous CABG	1.31	0.84–2.03	0.24	1.83	1.36–2.46	<0.001
Atrial fibrillation	1.74	1.33–2.49	0.002	1.91	1.51–2.40	<0.001
Renal impairment	1.54	0.95–2.5	0.08	1.30	0.92–1.84	0.13
Presentation						
NSTEMI	1.18	0.67–2.10	0.57	1.23	0.80–1.90	0.35
UAP	1.23	0.67–2.25	0.50	1.61	1.03–2.50	0.03
Elective	0.69	0.39–1.21	0.20	1.13	0.74–1.71	0.58
**Procedural**						
In-stent restenosis	1.29	0.70–2.37	0.42	1.55	1.06–2.27	0.03
LAD	-	-	-	1.29	1.03–1.61	0.03
Bypass	-	-	-	1.97	1.22–3.18	0.01
Multivessel disease	1.33	1.0–1.76	0.05			
Stent diameter (per unit)	-	-	-	0.77	0.63–0.93	0.01
Aspirin use at discharge	-	-	-	0.70	0.41–1.20	0.20
P2Y12 inhib. at discharge	-	-	-	0.50	0.27–0.93	0.03

MI—myocardial infarction; PCI—percutaneous coronary intervention; CABG—coronary artery bypass grafting, UAP—unstable angina pectoris; CI—confidence interval.

## Data Availability

The data underlying this article will be shared upon reasonable request to the corresponding author.

## Supplementary Materials

The following supporting information can be downloaded at: https://www.mdpi.com/article/10.3390/jcm12041684/s1, Table S1: Clinical Outcomes at 2 and 3 years following Unplanned Readmission within 12 months of PCI after propensity matching.
