# Supplementary material for: Early vs. Late Readmission following Percutaneous Coronary Intervention: Predictors and Impact on Long-Term Outcomes
Source: J Clin Med. 2023 Feb 20;12(4):1684. doi: 10.3390/jcm12041684 (PMC9958941; doi:10.3390/jcm12041684)
Supplement: Supplementary file 1 [file jcm-12-01684-s001.zip › jcm-2110144-supplementary.pdf]

Supplementary Data

Table S1. Clinical Outcomes at 2 and 3 years following Unplanned Readmission within 12 months of PCI after propensity matching

Outcome n (%)	Overall	No Readmission	Unplanned Readmission	P-value
	16,631	15,209	1,422	
**2 year Follow-up**				
**Followed up events**	**10,997**	**9,887**	**1,110**	
Death	1.6% (179)	1.3% (133)	4.1% (46)	<0.001
Myocardial Infarction	0.6% (69)	0.5% (53)	1.4% (16)	<0.001
TVR	0.5% (54)	0.5% (45)	0.8% (9)	0.11
TLR	0.7% (75)	0.6% (60)	1.4% (15)	0.004
MACE (composite)	2.7% (294)	2.3% (225)	6.2% (69)	<0.001
Procedures lead to any readmission	16.5% (1,815)	15.5% (1,535)	25.2% (280)	<0.001
Any unplanned readmission	6.0% (659)	5.4% (533)	11.4% (126)	<0.001
**3 year Follow-up**				
**Followed up events**	**8,483**	**7,608**	**875**	
Death	1.6% (133)	1.5% (117)	1.8% (16)	0.51
Myocardial Infarction	0.6% (54)	0.6% (48)	0.7% (6)	0.85
TVR	0.4% (32)	0.3% (26)	0.7% (6)	0.12
TLR	0.6% (47)	0.6% (45)	0.2% (2)	0.17
MACE (composite)	2.5% (210)	2.4% (183)	3.1% (27)	0.22
Procedures lead to any readmission	14.5% (1,226)	14.0% (1,068)	18.1% (158)	0.001
Any unplanned readmission	5.0% (423)	4.7% (357)	7.5% (66)	<0.001

TVR-Target vessel revascularization; TLR- Target lesion revascularization; MACE-Major adverse cardiovascular events.
